# Similarity measures and attribute selection for case-based reasoning in transcatheter aortic valve implantation

**DOI:** 10.1371/journal.pone.0238463

**Published:** 2020-09-03

**Authors:** Hélène Feuillâtre, Vincent Auffret, Miguel Castro, Florent Lalys, Hervé Le Breton, Mireille Garreau, Pascal Haigron

**Affiliations:** 1 Univ Rennes, CHU Rennes, Inserm, LTSI–UMR 1099, Rennes, France; 2 Therenva, Rennes, France; Vietnam National University, VIET NAM

## Abstract

In a clinical decision support system, the purpose of case-based reasoning is to help clinicians make convenient decisions for diagnoses or interventional gestures. Past experience, which is represented by a case-base of previous patients, is exploited to solve similar current problems using four steps—retrieve, reuse, revise, and retain. The proposed case-based reasoning has been focused on transcatheter aortic valve implantation to respond to clinical issues pertaining vascular access and prosthesis choices. The computation of a relevant similarity measure is an essential processing step employed to obtain a set of retrieved cases from a case-base. A hierarchical similarity measure that is based on a clinical decision tree is proposed to better integrate the clinical knowledge, especially in terms of case representation, case selection and attributes weighting. A case-base of 138 patients is used to evaluate the case-based reasoning performance, and retrieve- and reuse-based criteria have been considered. The sensitivity for the vascular access and the prosthesis choice is found to 0.88 and 0.94, respectively, with the use of the hierarchical similarity measure as opposed to 0.53 and 0.79 for the standard similarity measure. Ninety percent of the suggested solutions are correctly classified for the proposed metric when four cases are retrieved. Using a dedicated similarity measure, with relevant and weighted attributes selected through a clinical decision tree, the set of retrieved cases, and consequently, the decision suggested by the case-based reasoning are substantially improved over state-of-the-art similarity measures.

## Introduction

Aortic stenosis (AS) is the most commonly occurring valvular heart disease [1], and its severity, and prognosis are diagnosed using echocardiography. The management of patients is performed by a multi-disciplinary team. This “heart team”, which consists in part of cardiologists, cardiac surgeons, imaging specialists, anesthetists and cardiovascular nursing professionals, have to consider several issues before making decisions [1,2]. The members of the heart team have to review the medical condition of the patient (e.g., risk score and comorbidity), the clinical features, the anatomy, and technical factors (e.g., valve morphology, porcelain aorta). According to clinical experience guidelines [1,3], the best treatment strategy is established based on a benefit-risk assessment. In the case of severe AS, two strategies are considered: surgical aortic valve replacement (SAVR) or transcatheter aortic valve implantation (TAVI).

TAVI was initially developed for patients who are not candidates for surgery or for high-risk patients [4]. In just over the 15 years since it development, the technique has been shown to be effective, revolutionizing the management of severe AS. TAVI is currently an alternative treatment for intermediate-risk and low-risk patients [5,6]. This technique is continuously being developed with the onset of novel clinical devices and recommendations, and this raises new and complex issues about procedure planning, the anticipation of complications and patients’ options to avoid futile gestures [7]. Options to be decided on include whether the approach taken would be the vascular access route or the valve prosthesis type. The patient-specific decision-making process, which is based on anatomical and clinical characteristics as well as clinicians’ own prior experience, raises difficulties related to the comprehension of available, useful and relevant data.

In this paper, a clinical decision support system (CDSS) [8] that relies on case-based reasoning (CBR) is introduced, with the goal of helping practitioners to make decisions about the TAVI procedure.

The main concept of case-based reasoning is to learn from previous experiences, even with a limited number of previous patient cases. This accumulated knowledge plays an essential role in decision making when facing new problems. The basic assumption of a CBR system is that similar cases should have similar solutions. CBR differs from other major artificial intelligence (AI) approaches, especially those that are based on learning process such as machine learning (ML), or other knowledge-based systems (e.g. rule-based reasoning—RBR) [9,10]. CBR learns from previously processed cases, and the knowledge is progressively acquired [11]. The learning process is more evolutive than ML methods that require a special training phase, which is applied once from large datasets, to make future predictions. While CBR uses specific knowledge in the form of previous experience (the solved cases in the case-base), RBR, which is considered as pattern matching, represents general knowledge through a set of rules (if-then statements) [9,10]. The increased knowledge and experience in CBR becomes an advantage for medical applications when devices or clinical guidelines are continuously developed.

A case is represented by a set of attributes, which are obtained from clinical data and which can have different types. The CBR is composed of four steps: *retrieve*, *reuse*, *revise*, and *retain* [9,11]. The retrieve step is mandatory and requires data processing to evaluate reliably the similarity between cases and to recover relevant past cases. The other steps are defined according to the application, and users may be required to make decisions on reuse, revision, and case retention after the application and evaluation of the proposed solution. CBR does not need a substantial database. Even if the case-base increases according to the intended use of the CBR, the case-base is maintained by keeping useful and relevant information [12,13].

CBR has already been applied in various domains, such as statistical quality control [14], chemical engineering [15], signal-interpreting systems [16], and health science [17–19]. In the medical domain, according to a survey [17,18], CBR systems have different applications, such as diagnosis [20–22], classification [23–25], tutoring [26], planning [27,28], and knowledge acquisition [29]. Most of these CBR applications have been developed for specific diseases. Gu et al. [30] proposed a CBR to improve the accuracy of breast cancer recurrence prediction, and Bentaiba-Lagrid et al. [31] reported an approach to classify mammography mass and thyroid diseases. Torrent-Fontbona et al. [32] developed a CBR, using a numerical solution as an output rather than predetermined class labels to quantify the bolus insulin dosage. CBR has also been recently used for medical image processing applications, e.g., to improve kidney tumor segmentation as reported by Marie et al. [33].

Recently, CBR systems have been developed using AI techniques. These hybrid CBR systems have been coupled with rule-based reasoning (RBR) [21,22], fuzzy logic [34], data mining [35], neural networks [36], and genetic algorithms (GAs) [17,20]. Such combinations have been reported for the different steps of the CBR. Recently, Homem et al. [37] used a partial reinforcement learning algorithm to learn cases and to perform case-based maintenance in the context of robot-soccer. Gu et al. [30] combined ensemble learning with CBR to explain breast cancer recurrence prediction. Saraiva et al. [22] used rule-based reasoning to improve the retrieve step in the diagnosis of gastrointestinal cancer.

CBR has recently been considered as a useful decision support system for the diagnosis of clinical questions [17,18]. It is suited to medical problems, where knowledge is continuously evolving and where cases include many features [17]. For treatment purposes, CBR has the advantage of providing similar historical cases in addition to predictions. These similar cases provide a large amount of relevant information for decision making about the current patient, such as procedure and patient outcome after several months.

The feasibility of designing CBR for TAVI has been previously reported in [38]. That work concentrated on the overall framework and its integration in the clinical workflow, but did not focus on investigating the similarity functions. A classical definition of a similarity measure was used, and only a simple representation of cases was considered. In the retrieve step, different techniques can be used to obtain similar cases. While the most common retrieval technique has been the nearest neighbour retrieval (*k*-NN), a few CBR systems have used inductive or knowledge-guided approaches [17,39–41]. The similarity measure has represented a decisive part in the context of nearest neighbour retrieval. Wilson and Martinez [42], Lesot et al. [43], and Choi et al. [44] presented different comparison studies about similarity measures that have been used in various applications (e.g., data mining, data analysis, or information retrieval). Other research works studied the similarity measures in CBR systems, such as studies by Liao et al. [45], Núñez et al. [46], Avramenko and Kraslawki [15], and more recently, Gu et al. [20]. These different studies emphasized that the types of different attributes representing a case influenced the performance of the similarity measure, as did their degree of importance and the consideration of missing values.

Our proposed approach focuses on defining a relevant similarity measure to retrieve similar past cases. Depending on to the decision to be made, different issues have been addressed when defining the similarity measure, such as the choice of metrics, the selection of attributes, their degree of importance, and their mode of combination. In the design of the hierarchical similarity measure, the experience and reasoning of the “heart team” have been incorporated by the building of a clinical decision tree (CDT).

In the remainder of this paper, a description of related works about similarity measure is presented. The characteristics of the CBR framework that are deployed for the planning of the TAVI procedure are then presented in detailed. Next, the new hierarchical similarity measure based on the CDT presented, as well as the criteria used for evaluation. Finally, the results are presented and discussed for a case-base of patients who underwent the TAVI procedure.

### Related work

To obtain similar cases in the retrieve step, similarity measures are generally computed using dissimilarity measures (Eq 1) [42,47]. Most of the CBR system used a similarity measure that is based on a generalised weighted distance metric (Eq 2). The dissimilarity measure *diss*(*C*_*c*_, *C*_*i*_) between the candidate case *C*_*c*_ and a past case *C*_*i*_ is computed using the weighted sum of the attribute differences and is in the range [0,1]. *w*_*a*_ corresponds to the weight of attribute *a*, and *d*(*C*_*c*,*a*_, *C*_*i*,*a*_) represents the distance between the attribute *a* in cases *C*_*c*_ and *C*_*i*_. *n* represents the number of attributes considered.

$$sim(C_{c},C_{i})=1-diss(C_{c},C_{i})$$ (1)

$$diss(C_{c},C_{i})=\frac{\sum_{a=a_{1}}^{a_{n}}w_{a}d(C_{c,a},C_{i,a})}{\sum_{a=a_{1}}^{a_{n}}w_{a}}$$ (2)

A variety of distance measures were available, such as the Minkowski, Camberra, Chebychev, Mahalanobis, Cosine, and Jaccard metrics [42–44,48]. A large number of CBR systems used the weighted Euclidean distance. Although most attributes are quantitative, the Euclidean distance and the other distance metrics are not suitable for all data types.

The Euclidean distance is more appropriate for continuous quantitative values. A few works [14,15] converted ordinal attributes to discrete values. An integer value was assigned to each category (for example, 1 for *Mild*, 2 for *Moderate* and 3 for *Heavy*.). Afterwards, the distance measure between these integer values could be used to compute their degree of similarity. However, this type of discretisation was not applicable or suitable for a few of the cases. The ratio between each category may be different, and this value inconsistent.

Another solution was to use a heterogeneous distance measure [20,42]. Wilson and Martinez [42] proposed a distance function, the heterogeneous Euclidean-overlap metric (*HEOM*,S1 Appendix), which used the overlap metric for qualitative (i.e., nominal) attributes and the normalised Euclidean metric for quantitative attributes. The weighted heterogeneous Euclidean-overlap metric (*WHEOM*) represented the *HEOM* metric, where each attribute is weighted.

Wilson and Martinez explained that the *HEOM* metric corresponded to a simplistic approach for the qualitative attributes [42]. Whether the values of the nominal and ordered attributes were quite similar or different, their contributions were equivalent owing to the binary process used in the distance computation. They proposed to use another metric, the value difference metric (*VDM*), which was introduced by Stanfill and Waltz [49], instead of the overlap metric. The heterogeneous value difference metric (*HVDM*) combined the benefits of the Euclidean distance and *VDM* on the quantitative and nominal attributes, respectively.

An increasing number of CBR systems have used the heterogeneous similarity measure with the Euclidean distance for the continuous quantitative attributes. However, they had a different approach for qualitative attributes. Sheraf-El-Deen et al. [21] and El-Fakdi et al. [38] used as a basis a weighted heterogeneous distance metric in their retrieve step (generalised weighted heterogeneous similarity measure–*GWHSM*), while Gu et al. [20,30] opted for the *WHVDM* metric. Guessoum et al. [50] determined the similarity between qualitative attributes by employing a similarity matrix built from expert knowledge. *GWHSM* [38] makes use of the Euclidean distance for numerical attributes and the Hamming distance for the categorical data (S1 Appendix). Attributes are discarded if the value is unknown in a case. Missing values do not play any part in the similarity measure.

In addition to the metrics formulation, the weight of the attributes has an important impact in case retrieval. Weighting and scaling were used to reflect the importance of attributes in decision-making. Several ways to establish weight have been reported. They were fixed thanks to expert knowledge [17], making them interpretable and not database dependent. Learning-based approaches, such as GAs [17,51] were also used to weight the attributes. However, some CBR systems assigned the same importance to each attribute [38]. The management of missing values is also an issue in the similarity measure, and different approaches have been proposed [45,52,53]. Some CBR systems [38] discard the attribute when a value is missing. Other approaches estimate the distance between two case attributes when at least one of them is missing [50] or tried to complete the voids directly in the case-base before using the CBR system.

### CBR framework in TAVI application

This section presents the CBR concept that is proposed for TAVI. From our perspective, the main goal of clinical CBR is to support the practitioner in decision-making. One of the first intentions of this clinical CBR is to integrate the reasoning of practitioners in the system. For TAVI, the decisions are related to the procedure characteristics: the implanted valve type, valve diameter, and type of planned access. In clinical routines, practitioners follow the guidelines and decision trees, which they would have developed through experience. Given the relevance of decision trees in the reasoning process, we choose to integrate them in the retrieve step, i.e., in the proposed similarity measure.

### Data and case definition

The dataset used in this paper was retrospectively constituted from patients included at the University Hospital of Rennes in the registry FRANCE TAVI. Patients provided written informed consent for the procedure and for the anonymous processing of their data. The registry was approved (NCT01777828) by the Institutional Review Board of the French Ministry of Higher Education and Research and by the National Commission for Data Protection and Liberties.

**Fig 1:**
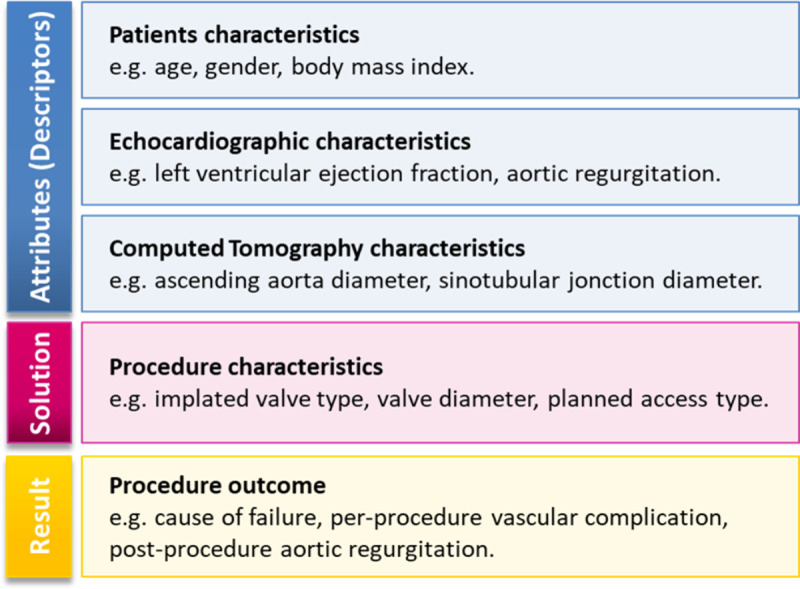
Three attribute categories of a clinical case in the TAVI database.

A case, i.e., a patient, which is the central notion in a CBR system, represents the experience of physicians. The set of past cases is used to build the case-base *CB*. Each case *C*_*i*_(*a*,*s*,*r*)∈*CB* is composed of three categories of data that are specifically collected during the aortic valve implantation (Fig 1):

- the description of the problem represented by a feature vector *a* = (*a*_1_, *a*_2_,…,*a*_*n*_), where *n* is the number of attributes (clinical attributes from patient characteristics and medical imaging such as the age or the diameter and calcification state of the aortic annulus),
- the solution *s* (procedure characteristics, such as the choice of the vascular access),
- the results *r* (procedure outcome, such as the procedure success, the annulus rupture, and the post-procedure aortic valve area).

S2 Appendix presents in detail the different data used in the clinical routine, and which was considered in the CBR module for TAVI application. These clinical attributes are used in different steps of the CBR process. Their similarities between different patients are exploited in order to propose a relevant solution for decision support. The input attributes acquired in the feature vector *a* can be of different types:

- continuous and discrete quantitative attributes such as diameter, area of the aortic annulus, and age,
- qualitative attributes that are ordered, called ordinal attributes, such as the tortuosity or the calcification of the different arteries,
- qualitative attributes that correspond to the Boolean category, such as the presence of calcification in the left ventricular outflow tract (LVOT).

### CBR solving cycle

The operation of CBR is based on human–machine cooperation. The reasoning system makes suggestions, but the user remains in control of the final decision. CBR thus makes use of the complementarity between the practitioner (reasoning and decision to take) and the machine (computation).

**Fig 2:**
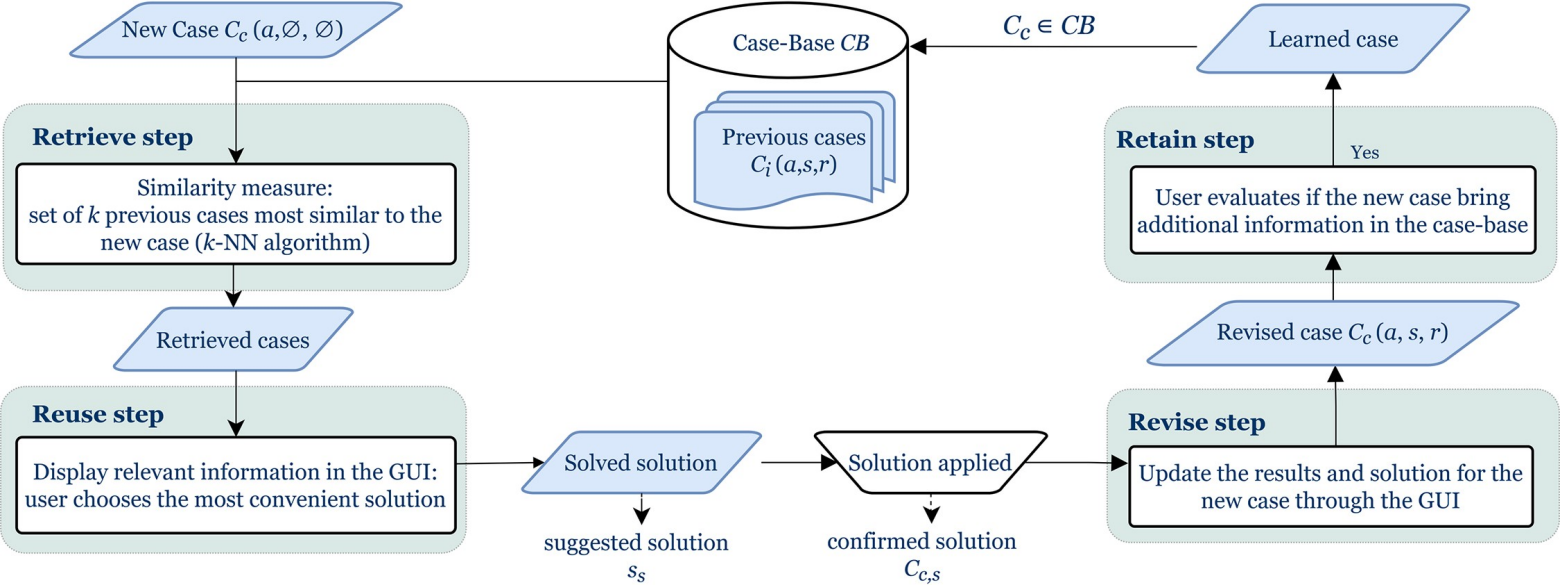
CBR steps.

The solution *C*_*i*,*s*_ of the past cases *C*_*i*_ stored in the case-base is already known. However, the new case *C*_*c*_(*a*,∅,∅), from which the CBR will be executed, is not in the case-base (*C*_*c*_∉*CB*) and its solution *C*_*c*,*s*_ is still unknown. Based on the four steps presented in the Fig 2, the goal of CBR is to support the physician to make the most suitable decision about the solution *C*_*c*,*s*_.

The retrieve step involves computing the similarity between cases to highlight the set of the most similar previous cases based on the *k*-NN algorithm. Using the graphical user interface (GUI), the user selects the value of *k* before launching the retrieve step. The design of the similarity measure, which include a clinical decision tree, is presented in detail in the following section.

In the reuse step, the CBR suggests a solution *s*_*s*_ from the set of *k* most similar cases. This step can be solved as a classification problem, where the class of the current case *C*_*c*_ has to be determined. Different methods [38,47] enable determination of the class, i.e. the solution, of the current case *C*_*c*_. As shown in Eq 3, a democracy voting weighted both by the distance and the rank of the similar cases is proposed. (*s*, *C*_*i*,*s*_) returns 1 if the solution of the past case *C*_*i*,*s*_ corresponds to *s*, and 0 otherwise. *rank*_*i*_ denotes the ranking of the past case *C*_*i*_∈*CB* in the set of *k* retrieved cases. It enables more weight to be assigned in the first similar cases for the class determination. *diss*(*C*_*c*_, *C*_*i*_) represents the distance value between the current candidate case *C*_*c*_ and a past case *C*_*i*_.

$$Vote(s)=\sum_{i=1}^{k}\frac{1}{diss(C_{c},C_{i})}({k+1-rank}_{i})(s,C_{i,s})$$ (3)

$$s_{s}=\arg\;\underset{s}{\max}(\mathrm{Vote}(s))$$

The results of the CBR for a case are displayed in a user-friendly interface to facilitate the relevant information derived from the set of *k* similar cases, and to enable the complete integration of the practitioner in the reasoning system. The most relevant attributes, such as the procedure outcomes, are displayed for each similar case. For each possible solution *s*, the corresponding *vote*(*s*) obtained in the reuse step is converted into a percentage (*vote*(*s*)×100/∑_*s*_*vote*(*s*)). It is then used to represent the level of confidence in the solution, and to allow the user to appreciate the reliability of the suggested solution.

The user participates directly in the two last steps. The clinician has evaluated and applied the suggested solution *s*_*s*_. This suggested solution of the current case *C*_*c*_ then becomes the confirmed solution *C*_*c*,*s*_.

In the revise step, the information about the solution *C*_*c*,*s*_ and the result *C*_*c*,*r*_ (i.e., the procedure outcomes) are incorporated into the current case *C*_*c*_(*a*,*s*,*r*) through the GUI.

In the retain step, if the user considers that the revised case provides relevant information, the retention of the case is performed through the GUI to update the case-base. The GUI allows the user to add a new solved case or to choose to remove a previous solved case in the case-base. The user can also add information about the follow-up of the patient. Thus, the CBR continuously acquires knowledge by learning from the cases that have already been processed. When a candidate case is retained, the associated retrieved cases are also memorized. As our work focuses on the retrieve and reuse steps, this information is not currently processed in the CBR, but it could be used to complete the learning process in a future version to automatically identify relevant cases and to strengthen the retain step, which is always under the user's control.

### Hierarchical similarity measure

The quality of the results given by the CBR system depends mainly on the definition and the performance of the similarity measure. The definition of a convenient similarity measure represents an important issue at the retrieval stage. The goal is to help the practitioner to make decisions about the vascular access, the type and the size of the prosthesis. Our approach relies on the definition of a dedicated metric from clinical attributes, which is available in the clinical database, combined with attribute selection and weight determination through CDTs.

### Clinical Decision Trees (CDTs)

**Fig 3:**
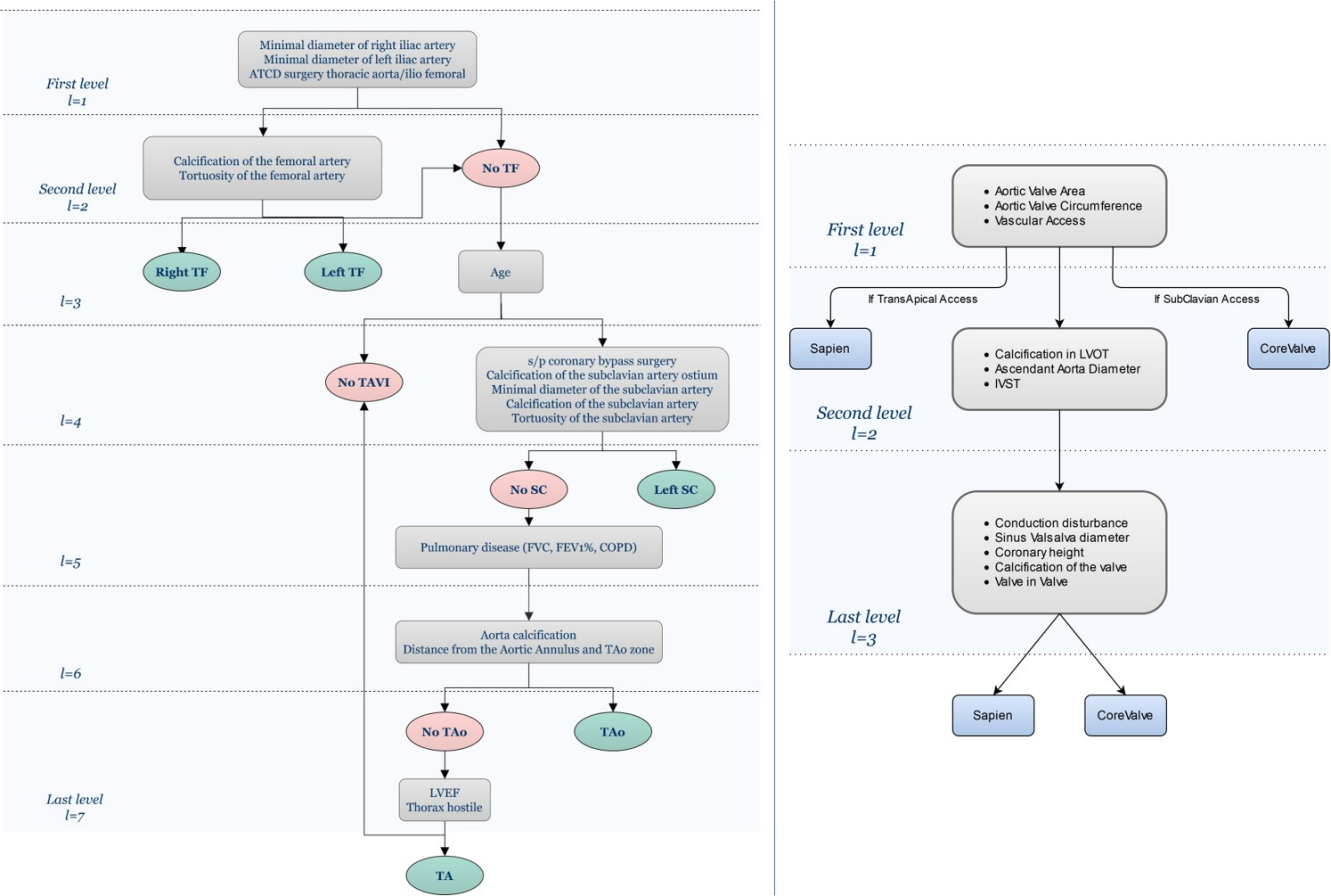
Attribute hierarchy in CDTs. Left: the CDT used in TAVI for the vascular access choice. Right: the CDT for the prosthesis choice (both type and size). TF: trans-femoral, SC: left trans-subclavian, TAo: trans-aortic, TA: trans-apical.

It is essential to consider relevant attributes in the similarity measure. According to the different decision levels, the attributes in the case-base do not have the same importance. From expert knowledge and the literature (guidelines [1–3], expert consensus [2], and medical papers [6]), the rules (which translate contraindications or preferences) and questions related to the decision making process have been highlighted. They can be separated according to the type of decision: which vascular access, which type and size of prosthesis. They have been represented using a CDT for each type of solution supported by the CBR (Fig 3). A few rules may change depending on the hospital and physician as well as the improvement of devices (e.g., prosthesis and catheter) and new guidelines. These differences can be easily considered in the CDTs that are used in the CBR.

With respect to the vascular access, the left and right trans-femoral accesses are used in most cases (more than 80% of the cases [2,6]). Then, the left trans-subclavian access or the trans-carotid access is preferred depending on the hospital centre. In the current case-base, the trans-carotid was not considered. The trans-aortic and trans-apical vascular accesses are increasingly infrequent because they are more invasive. Further, they are highly contraindicated for elderly patients. Specific decision rules and conditions must be respected in hierarchical order for each type of vascular access. For example, for the trans-femoral access, the diameter of the arteries is first examined to determine if these two accesses may be used during the intervention. If the diameters of the left and right arteries are adequate, the tortuosity and the calcification are then checked. In addition, previous diseases on femoral arteries represent a contraindication for use of this vascular access. A previous aneurysm or thrombus means that arteries are frailer, and the risk of dissection is higher during the intervention.

The type and size of the prosthesis are linked, and they both represent the characteristics of the device. The different available prostheses do not have the same range of sizes. For example, the Medtronic CoreValve Classic exists in 23, 26, 29, and 31 mm while the Edward Sapien XT is available in 20, 23, 26, and 29 mm. As mentioned above, decision rules can be highlighted with respect to these questions. The most important attributes for the size decision are the dimensions (area and diameter) of the aortic annulus. In terms of prosthesis type, the choice depends on the vascular access that was selected previously. For example, if the trans-apical access is used, the Edward Sapien XT valve would be implanted, and operators would use the Medtronic CoreValve for the left trans-subclavian access. When both prosthesis types can be deployed, operators often choose according to their practice and preference, providing that there is no contraindication. The authors in [38] separately treated the choice of prosthesis type and size. In our approach, we propose only one decision for the prosthesis, as type and size are closely related. In this way, the considered CBR is constrained, thus avoiding the proposal of an incoherent combination of type and size of prosthesis.

Even though the selected attributes are relevant in terms of decision-making, they do not have the same influence depending on their level in the CDT. Attributes in the root present a higher importance in the decision-making process than attributes near the leaves. For instance, for the decision related to the vascular access, the minimum diameters of the iliac arteries are among attributes considered in the first level of the CDT because trans-femoral access is first preferred clinically.

### Distance metric

The clinical decision-making process is inherently hierarchical, and the reasoning and knowledge of the clinician are transposed through the CDT. Even if all the attributes of the CDT can be considered (weighted) using classical similarity measures, they can hardly be used to formulate the CDT hierarchy which implies a non-linear combination of the distance relative to the attributes. The proposed hierarchical heterogeneous similarity measure H_WHSM (Eq 4) also exploits the hierarchy of the CDT to select relevant attributes and to weight them.

$$H\_WHSM(C_{c},C_{i})=1-{diss}_{L}(C_{c},C_{i})$$ (4)

$${\mathrm{with}\;diss}_{l}(C_{c},C_{i})=\frac{\sum_{a_{l}=a_{1,l}}^{a_{n,l}}w_{a_{l}}d(C_{c,a_{l}},C_{i,a_{l}})}{\sum_{a_{l}=a_{1,l}}^{a_{n,l}}w_{a_{l}}}\mathrm{and}\;l\in [1,L]$$

*l* corresponds to the current level in the CDT, and *L* is the height of the CDT. *C*_*i*_ with i∈[0,m2l] represents a retained case in the case-base, and *m* is the total number of cases in the case-base. $d(C_{c,a_{l}},C_{i,a_{l}})$ is calculated for each attribute *a* that is available in the CDT, and it is defined according to the type of attribu (Eq 5).

$$d\left(C_{c,a_{l}},C_{i,a_{l}}\right)=\{\begin{array}{l} d_{E}\left(C_{c,a_{l}},C_{i,a_{l}}\right)\;\mathrm{if}\;C_{c,a_{l}}\mathrm{and}\;C_{i,a_{l}}\;\mathrm{are}\;\mathrm{quantitative}\\ d_{H}\left(C_{c,a_{l}},C_{i,a_{l}}\right)\;\mathrm{if}\;C_{c,a_{l}}\mathrm{and}\;C_{i,a_{l}}\;\mathrm{are}\;\mathrm{binary}\\ d_{o}\left(C_{c,a_{l}},C_{i,a_{l}}\right)\;\mathrm{if}\;C_{c,a_{l}}\mathrm{and}\;C_{i,a_{l}}\;\mathrm{are}\;\mathrm{ordinal}\\ d_{M}\left(C_{c,a_{l}},C_{i,a_{l}}\right)\;\mathrm{if}\;C_{c,a_{l}}\;\mathrm{or}\;C_{i,a_{l}}\;\mathrm{are}\;\mathrm{missing} \end{array}$$ (5)

$$\mathrm{with}\;d_{E}\left(C_{c,a_{l}},C_{i,a_{l}}\right)=\frac{\left|c_{c,a_{l}}-c_{i,a_{l}}\right|}{range_{a}}$$

$$d_{H}(C_{c,a_{l}},C_{i,a_{l}})=\left\{ \begin{array}{c} 0,\;if\;C_{c,a_{l}}=C_{i,a_{l}}\\ 1,\;if\;C_{c,a_{l}}\neq C_{i,a_{l}} \end{array}\right.\;for\;C_{c,a_{l}},C_{i,a_{l}}\in \{yes,\;no\}$$

$$d_{O}(C_{c,a_{l}},C_{i,a_{l}})=O[C_{c,a_{l}}][C_{i,a_{l}}]$$

$$d_{M}(C_{c,a_{l}},C_{i,a_{l}})=0,5$$

The Euclidean distance $d_{E}(C_{c,a_{l}},C_{i,a_{l}})$ is computed for quantitative attributes, and the Hamming distance $d_{H}(C_{c,a_{l}},C_{i,a_{l}})$ is used for binary attributes. For each type of ordinal data, a distance matrix $d_{O}(C_{c,a_{l}},C_{i,a_{l}})$ is built according to expert knowledge. This matrix converts the distance between two ordinal attributes to a quantitative value, which is normalized within the range [0,1]. The matrix determines how two attribute values are dissimilar according to the decision to be make. The categories that are considered in an ordinal attribute do not linearly qualify its influence on decision-making. Depending on the attribute, the decision can be influenced in very different proportions when the grade goes from *Mild* to *Moderate* or when it goes from *Moderate* to *Heavy*. This is why the distance transcribing the difference between two grades is assessed empirically by an expert clinician. Table 1 presents an example of the distance matrix used for the attribute relative to calcification. The distance between the attribute *Mild* and *Moderate* is 0.2, i.e., $d_{O}(C_{c,a_{l}},C_{i,a_{l}})=0.2$ with $C_{c,a_{l}}=Mild$ and $C_{i,a_{l}}=Moderate$. In terms of decision making, an artery with no calcification is quite similar to an artery with mild calcification. Even if the decision is different, the grades *Heavy* or *Massive* are also considered close. The distance value transcribing the gap between *No* and *Mild* is lower than the one transcribing the gap between *Moderate* and *Heavy*. Moreover, an online approach [50] is used to manage the missing value, if necessary. The neutral approach was chosen, which gives directly the value 0.5 as distance *d*_*M*_ between attributes.

**Table 1:** Example of distance matrix O used for the attribute calcification (ordinal data).

	No	Mild	Moderate	Heavy	Massive
**No**	0	0.15	0.5	0.90	1
**Mild**	0.15	0	0.2	0.5	0.7
**Moderate**	0.5	0.2	0	0.2	0.5
**Heavy**	0.90	0.5	0.2	0	0.15
**Massive**	1	0.7	0.5	0.15	0

The expression of the metrics *diss*_*l*_ constituting *H_WHSM* (Eq 5) is adapted according to each level *l* of the CDT. The different evaluation steps resulting in the complete metric *diss*_*L*_ are presented in detail in *Algorithm 1*. Starting with the first level of the CDT (Fig 3), only the most relevant attributes *a*_*l*_ = (*a*_1,*l*_,*a*_2,*l*_,…,*a*_*n*,*l*_), with *l* = 1, are considered in the evaluation of *diss*_*l*_. According to the computed distance value, a selection of past cases is made to keep only half of the most similar ones. The next levels of CDT are then considered. For each level, the distance metric *diss*_*l*_ is updated by considering the attributes present both in the previous levels and in the current level *l* of the CDT. At the end of the process, only the *m*/2^*l*^ most similar cases are retained and the distance *diss*_*L*_ enables the set of retrieve cases to be obtained.

The weighting scheme of *H_WHSM* makes use of the CDT. As it is expert knowledge-driven, it relies on the hierarchy of the attributes in the CDT. The attributes in the first level of the CDT, such as the *aortic valve area* for the type and size of the prosthesis (Fig 3), have more importance in the decision-making process than attributes in the other levels. The weights $w_{a_{l}}$, which are normalised within the range [0,1], are computed according to the attribute level *l*_*a*_ in the CDT, while considering the total number of levels *L* (Eq 6).

$$w_{a_{l}}=\left(L-l_{a}+1\right)/L,\;\mathrm{with}\;l_{a}\in \left[1,L\right]$$ (6)

***Algorithm 1*: *Computation of the similarity measure H_WHSM***

***Input*:** the initial case-base *CB*_*0*_, a case *C*_*i*_∈*CB*_*0*_ with *i*∈[*0*,*m*], and *m* is the total number of previous cases, *C*_*c*_ is the current candidate case, which is the CDT of *L* levels.

***Output*:** *diss*_*L*_(*C*_*c*_,*C*_*i*_) with *C*_*i*_∈*CB*_*L*−*1*_, and consequently *H*_*WHSM*(*C*_*c*_,*C*_*i*_)

**begin**

**1:** For each level *l*∈[*1*,*L*] of the CDT do

**2:**    *a*_*l*_ = (*a*_*1*,*l*_,*a*_*2*,*l*_,…,*a*_*n*,*l*_) // Attribute selection: to get all attributes in the CDT from level 1 to *l*

**3:**    Compute the weight $w_{a_{l}}$ of each attribute *a*_*l*_ according to Eq 6

**4:**    For each case *C*_*i*_∈*CB*_*l*−*1*_ with *i*∈[*0*,*m*/2^*l*^] do

**5:**        Compute *diss*_*l*_(*C*_*c*_,*C*_*i*_) //Eq 4

**6:**  End for each

**7:**    If *m*/*2*^*l*^>*20* and *l*≠L //the case-base cannot have less than 20 cases

**8:**        Keep in *CB*_*l*_ only half of the most similar cases *C*_*i*_ with *i*∈[*0*,*m*/*2*^*l*^] //Case selection

**9:**    End if

**10:** End for each

**11:** Compute *H*_*WHSM*(*C*_*c*_,*C*_*i*_) //Eq 4

**end**

### Evaluation approach

For evaluation purposes, the hierarchical weighted heterogeneous similarity measure *H_WHSM* is compared with two state-of-the-art similarity measures, which are presented in detail in S1 Appendix. In *HEOM*, no attribute selection and weighting scheme are performed [42]. *GWHSM* enables attributes to be weighted, even though they were set to 1 in the results reported in [38]. To compare the similarity measure based on expert knowledge and the CDT, a learning-based approach is used as a weighting scheme in *GWHSM_GA*. Using a GA to learn attribute weights; no prior expert knowledge is integrated in this last approach. GAs have already been adopted successfully in several CBR systems for weight determination [20,51]. In this approach, a floating-point chromosome representation is used to represent an individual. Each individual of the population in the GA represents a particular weight of the attributes of the similarity function. To calculate the fitness of each individual, the leave-one-out cross validation technique is employed. The average performance obtained using the weights for the similarity function is calculated by repeatedly removing a case with a known solution from the case base, the so-called target case, retrieving the most similar case from the remaining cases in the case base and comparing the solution of the retrieved case with the actual solution of the target case. The fitness function is defined as the precision (*TP*/(*TP*+*FP*)) of the number of solutions that are correctly proposed. The used evolutionary operators are crossover, mutation, and elitism (elitist selection). The best weight determination was obtained with the following configuration. The crossover rate is 0.75 and the mutation rate is 0.20. The number of generations that are used in the GA is 300. The proposed GA uses the roulette-wheel selection method. Elitism is used, and ensures that individuals in the top of 30% with respect to their fitness are taken to the next generation.

These three similarity measures imply different combinations related to the weighting scheme and attribute selection, and they are summarized in Table 2.

**Table 2:** Overview of the similarity measures.

Similarity measure	Attribute selection	Attribute weight	Case selection
*H_WHSM*	Yes—CDT	Yes–CDT Level and Hierarchical process	Yes
*HEOM*	No	No	No
*GWHSM_GA*	Yes	Yes–GA	No

To evaluate the similarity measure performance through cross validation, two evaluation criteria were considered. The first way to evaluate the performance of the similarity measure was to analyse the set of *k* similar cases that are obtained after the retrieve step: the retrieve-based criterion. For each candidate case *C*_*c*_, it is expressed as the number of cases with the correct solution *C*_*c*,*s*_ among the set of *k* retrieved cases.

Another way to evaluate the performance of the similarity measure was to analyse the correctness of the decision suggested by the CBR at the end of the reuse step: the reuse-based criterion. From the set of *k* most similar cases obtained through a given similarity measure, only one suggested solution was highlighted owing to Eq 3. The candidate case *C*_*c*_ was assumed to have been correctly classified when the suggested solution *s*_*s*_ was the same as the confirmed solution *C*_*c*,*s*_, i.e., the one that has been applied during the intervention.

The evaluations were performed on a real case-base of patients who underwent a TAVI procedure. To analyse the influence of the case-base content on the results, additional cases were also generated from the real data. The data augmentation process was used to double the size of the case-base with the generated cases. From a real case *C*_*i*_(*a*,*s*,*r*)∈*CB*, where *CB* is the real case-base, all attributes *C*_*i*,*a*_ describing the problem were modified to obtain the generated case. The solution *C*_*i*,*s*_ and result *C*_*i*,*r*_ were not changed. The distribution of generated cases remains the same as in real cases. The value of attributes resulting from the measurement was randomly modified to be consistent with the solution of the case. For instance, the area of the aortic valve has been specified in the range recommended by the device manufacturer (Instruction For Use) for a given prosthesis size. Other quantitative attributes, such as the age, weight, and height (and consequently the body mass index and body surface area), were also randomly modified until +/- 10% while respecting a clinically coherent interval. Categorical attributes had their value randomly modified with the upper or lower grade (or there were left identical).

## Results

**Fig 4:**
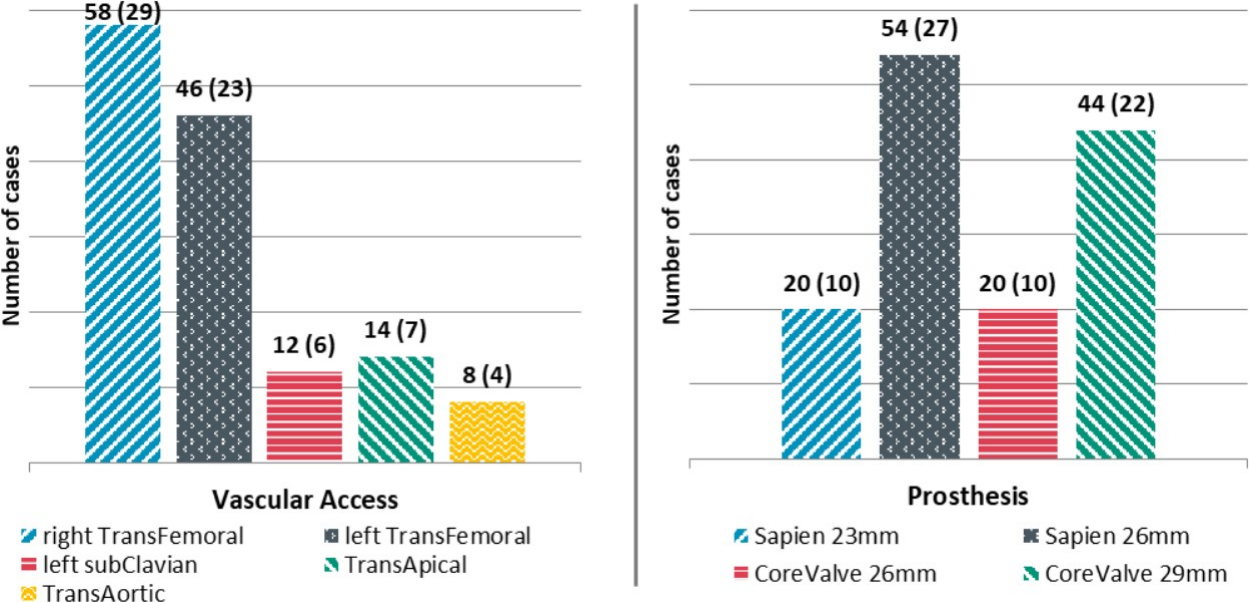
Distribution of cases in the augmented case-base according to the decision type (vascular access and prostheses). The number in bracket represents the number of real cases.

The real case-base that was used for the evaluation is composed of patients who underwent a TAVI procedure. For all patients, the attributes used in the CBR (patient and procedure characteristics) were directly obtained from data routinely available in clinical routines (S2 Appendix). There was no missing value in the dataset. Fig 4 shows the distribution of the 138 cases in the augmented case-base according to the two clinical decisions that were considered: the vascular access and the prosthesis (both type and size). Five vascular accesses are represented in the case-base. The number of cases with trans-femoral access (both the right and left sides) is consistent with that in the literature (around 80% [2,6,7]). Four combinations of prostheses are available: the *Edwards Sapien XT* in 23 mm and 26 mm, and the *Medtronic CoreValve* in 26 mm and 29 mm, respectively.

**Fig 5:**
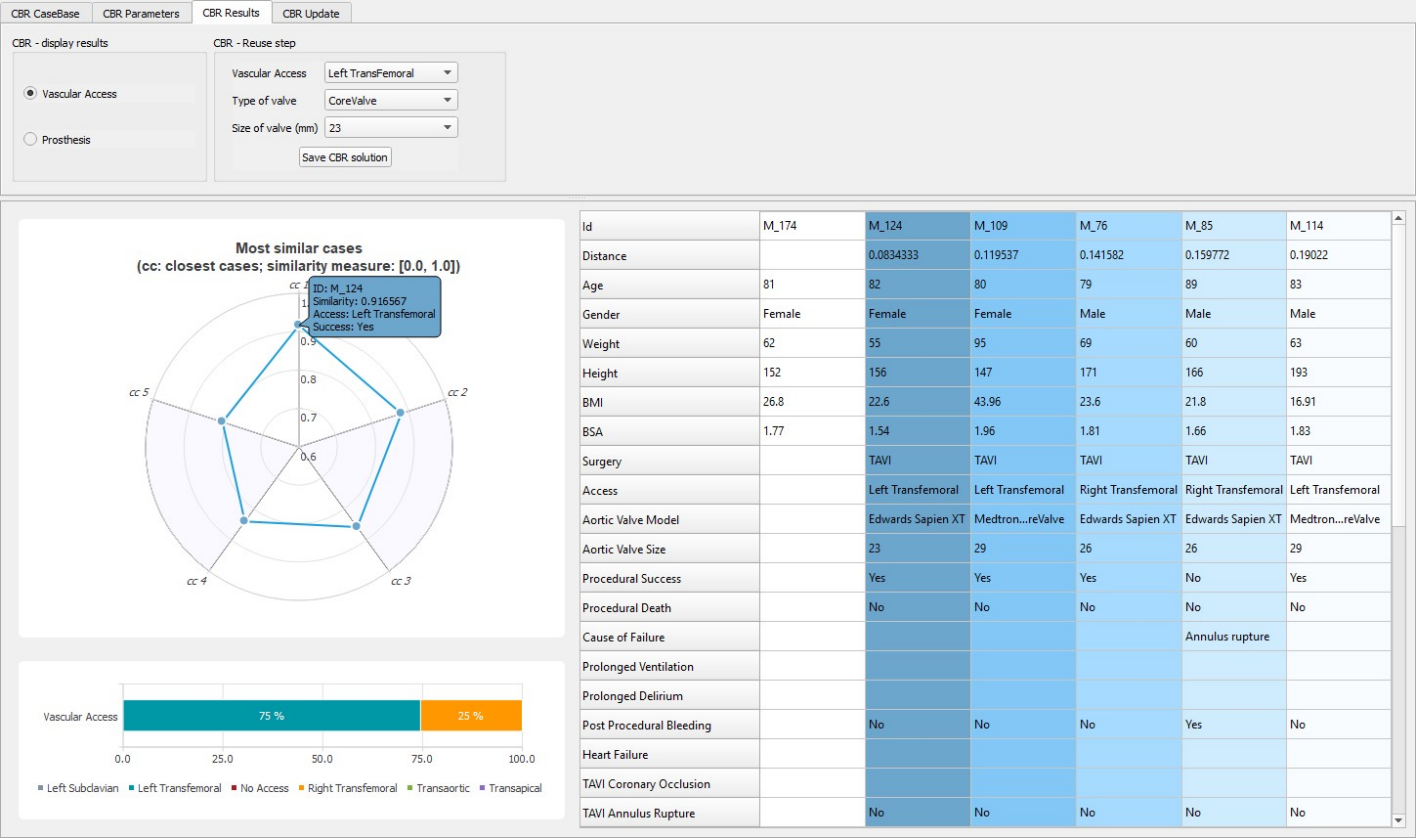
GUI screenshot of a vascular access result with *H_WHSM* and *k = 5*.

After retrieving the set of similar cases, the CBR software displays the relevant information for decision making in the form of charts and tables (Fig 5). First, the *k* similar cases are transcribed with relevant attributes in a table on the right of the GUI. They are sorted according to their distance with the candidate cases. These distances are also shown in the polar chart. The suggested solution, which is computed using the Eq 3 in the reuse step, is presented as the higher percentage in the bar chart, and represents the confidence in solutions suggested (already applied in the set of past cases). Both suggested solutions with respect to vascular access and prosthesis are computed by the CBR, and can be displayed in the GUI according to the selection made by the user (radio button at the top-right).

### Retrieve-based criterion

The first results relate to the global behaviour of the similarity measures when only the most similar case (*k* = 1) is retrieved. The similarity measure *H_WHSM* introduced in this work was compared using a leave-one-out cross validation, with the two state-of-the-art similarity measures: *HEOM* and *GWHSM_*GA (Table 2).

**Fig 6:**
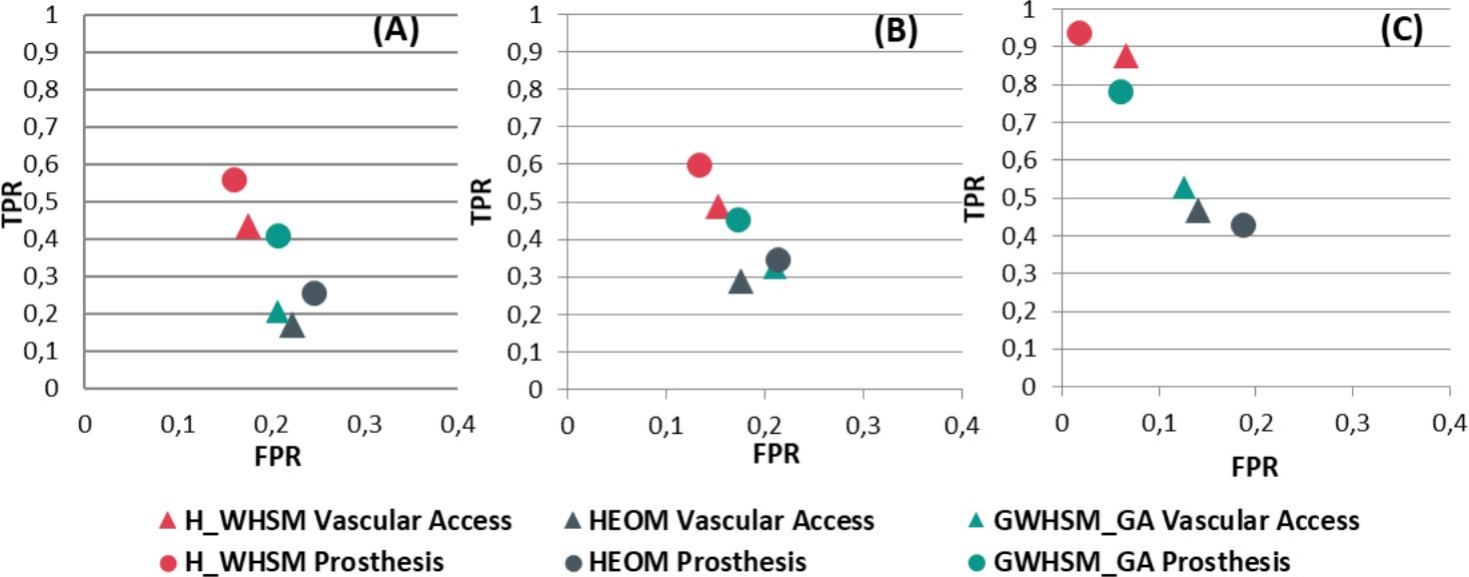
True positive rate (TPR) and false positive rate (FPR) for similarity measures according to the two decisions when *k = 1*. The leave-one-out cross validation results are reported for real cases (A) and generated cases (B), and for both real and generated cases (C).

Fig 6 shows the true positive rate (TPR) and the false positive rate (FPR) obtained in the leave-one-out cross validation for the vascular access and prosthesis decisions. The similarity measures are evaluated for the three case-bases that containing the real cases, the generated cases, and both cases (global case-base). For all case-bases, the best results were obtained with the hierarchical similarity measure *H_WHSM*, which surpass the state-of-the-art similarity measures. With the global case-base, the TPR reaches 0.94 for the prosthesis choice, and almost 0.9 for the vascular access decision.

**Fig 7:**
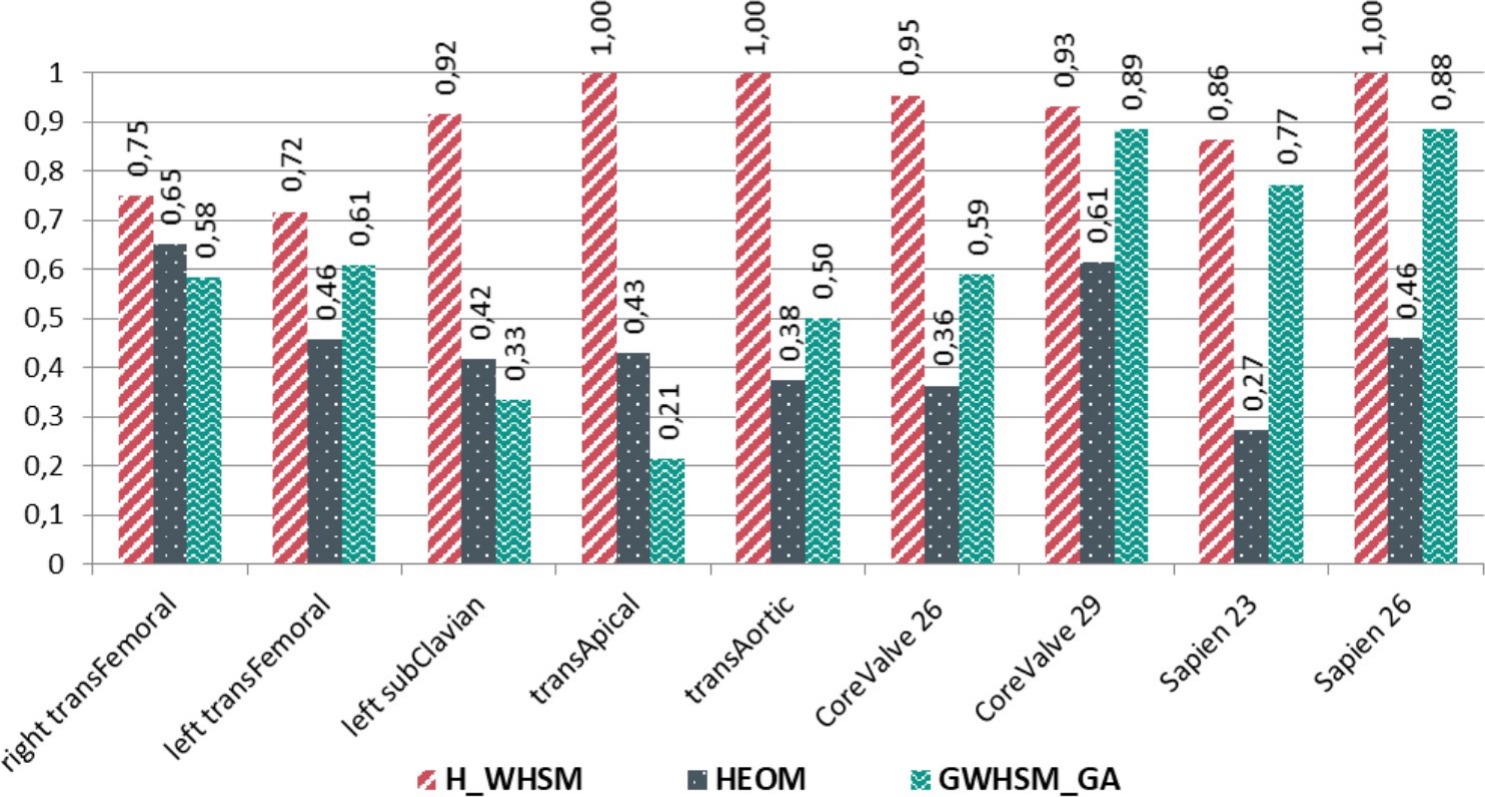
Sensitivity of similarity measures when one similar case is retained (*k = 1*) for the different solutions, computed from the leave-one-out cross validation on the global case-base.

**Fig 8:**
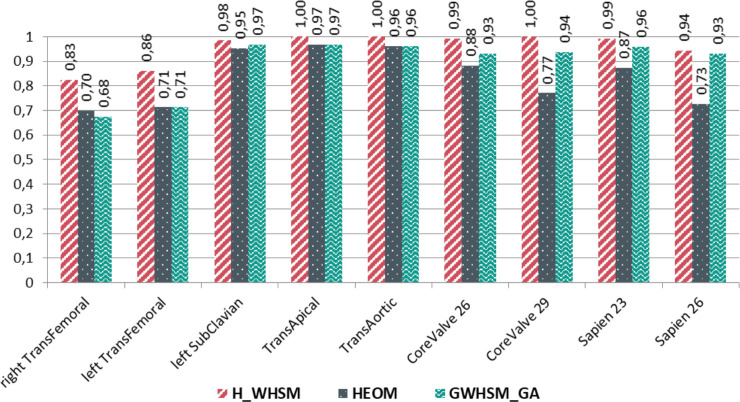
Specificity of similarity measures when one similar case is retained (*k = 1*) for the different solutions, computed from the leave-one-out cross validation on the global case-base.

In the next section, the CBR performance is examined using each possible solution available in the global case-base. Figs 7 and 8 show the specific results for the three similarity measures: *H_WHSM*, *HEOM*, and *GWHSM_GA*. The sensitivity and specificity of the similarity measures are computed for each possible solution when only one similar case is retrieved (*k* = 1). We assume that this most similar case has a higher probability to have the correct solution. We observe that the sensitivity value obtained with *HEOM* and *GWHSM_GA* is low for some solutions, such as the trans-apical access or the prosthesis Medtronic CoreValve 26mm. For *H_WHSM*, the specificity values are always higher than those obtained with the two state-of-the-art similarity measures. It should also be noted that lower specificity and sensitivity values are obtained for the trans-femoral access (both the right and left sides). When the trans-femoral access is considered as a single access irrespective of the side, the sensitivity and specificity of *H_WHSM* increase and reach 0.98 and 0.97, respectively, for the global case-base.

As the value of *k* can be chosen by the user, the following results describe the behaviour of the similarity measures when its value increases in the range *k*∈[0;7]. In these results, the maximum number of most-similar cases (k = 7) is set to 10% of the total number of real cases. A higher value could be chosen, which can lead to a less accurate suggested solution. In addition to representing a large amount of information, the retrieval of too many cases would be unnecessary and would distort the user’s decision. By setting the maximum value of k to 10% of the total number of real cases, which is assumed to be an upper limit, its impact on the results can be determined. Because *HEOM* gives almost the worst result, as shown previously, only *GWHSM_GA*, which also selects and weights the attributes, is kept for comparison purposes. Hereafter, the evaluation is performed using a cross validation with the real case-base as the training dataset and the generated case-base as the testing dataset. A candidate case *C*_*c*_ is now considered as correctly classified when the correct solution *C*_*c*,*s*_ appears at least once among the *k* retrieved cases. This analysis represents a consistent indicator of performance as the final decision is left to the user.

**Fig 9:**
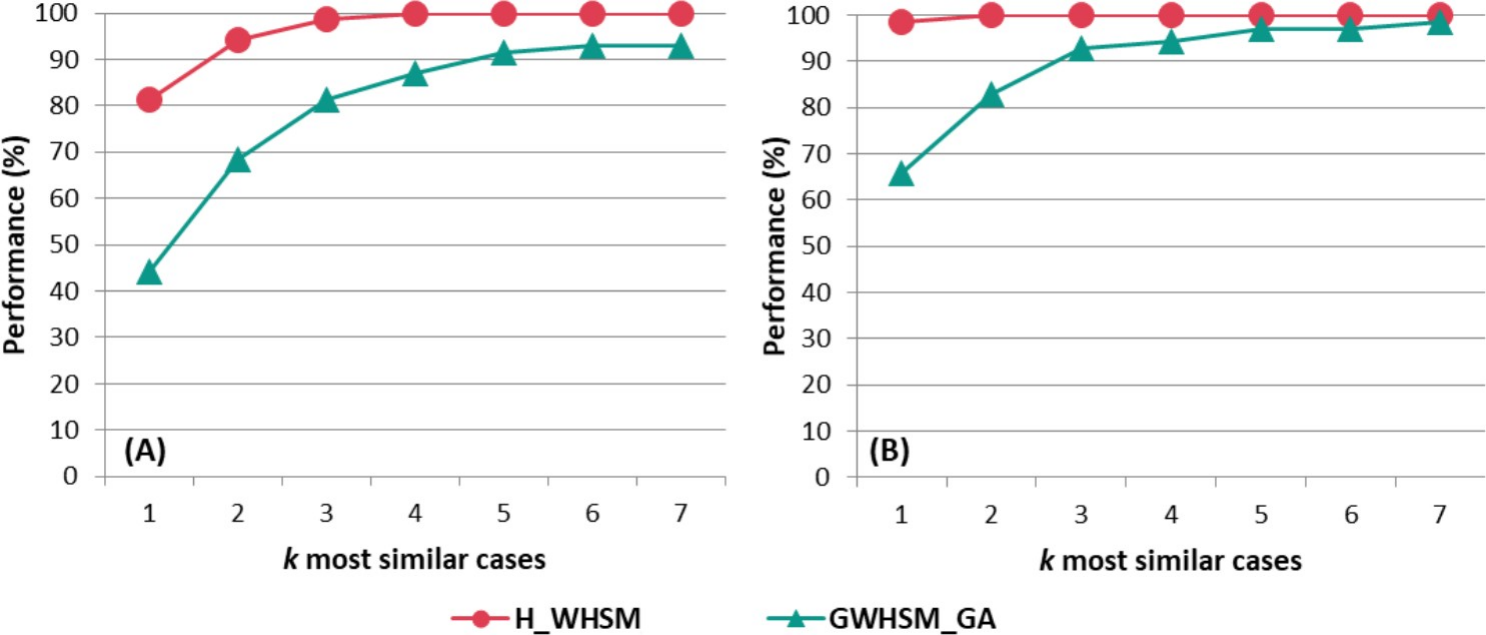
Percentage of cases where the correct decision appears at least once into the *k* most similar cases for (A) the vascular access and (B) prosthesis choice.

Fig 9 describes the percentage of cases that are correctly classified when *k*∈[1;7]. The performance increases significantly with the value of *k* reaches 90−100% for each similarity measure. A sharp increase is seen between the lowest values of *k*. For instance, there is a gap of 20% to 40% between *k* = 1 and *k* = 3, with the exception of *H_WHSM*, which already exhibits a performance close to 100% for the prosthesis choice when *k = 1*. For both decisions, the proposed measure *H_WHSM* improves the results for all *k* values. For the highest values of *k* in the prothesis choice decision, the two measures present a similar performance. However, compared to *GWHSM_GA*, *H_WHSM* enables a better set of *k* retrieved cases to be obtained.

### Reuse-based criterion

**Fig 10:**
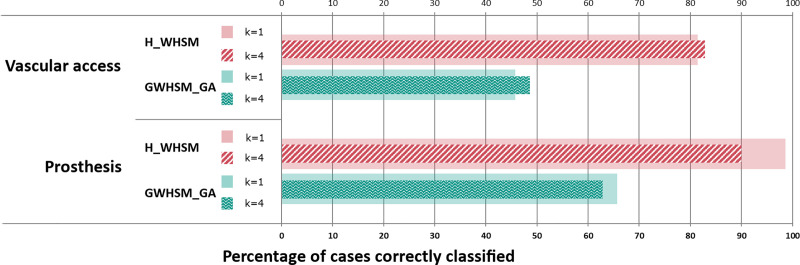
Percentage of suggested solutions that are correctly classified obtained for both decisions and two values of *k* with real cases as the training set and generated cases as the validation set.

The reuse-based criterion is based on the suggested solution given by Eq 3. Fig 10 describes for *k* = 1 and *k* = 4 the percentage of cases that are correctly classified according to the solution that is suggested for the vascular access and the prosthesis choice decisions. As was the case previously, the evaluation was performed, using a cross validation. The real cases are used to constitute the case-base and the training dataset. The generated cases constitute the validation dataset. As previously highlighted, the performance of *H_WHSM* gives the best proportion of cases correctly classified for the different values of *k*. Compared with *GWHSM_GA*, there are significant differences between the percentages of cases that are correctly classified. When the four most similar cases are selected (*k* = 4) for the prosthesis choice, 90% of the suggested solutions are correct for *H_WHSM* as opposed to 63% for *GWHSM_GA*. The same trend can be observed with *k* = 1. Results show that the best choice for k is not the same between vascular access and prosthesis choice. Overall, the value of *k* has little influence with respect to only the suggested solution, but it increases information about similar cases that are provided to the user through the GUI.

## Discussion

In order to support clinical decisions pertaining to vascular access and prosthesis choices in TAVI, we focused on the similarity measure which is a key component of the CBR. We examined the importance of considering CDTs and the selection of relevant attributes.

A weighted hierarchical similarity measure *H_WHSM* was proposed, and compared with two state-of-the-art similarity measures. These three similarity measures have different characteristics. Because the case-base did not have incoherent attributes (S2 Appendix), *HEOM* was considered as the basic similarity measure, using all the attributes of the case-base without weighting. Nevertheless, although the attributes were all related to the medical problem, they were not specifically linked to one decision. *GWHSM_GA* was used as a weighted similarity measure to select decision related attributes, even though results were initially reported with the weight fixed to 1 [38]. In our study, a GA was used to tune the weights that were allocated to the different attributes. The proposed similarity measure *H_WHSM* included a CDT in its definition. This hierarchical approach exploited the CDT to select the most similar cases progressively as well as to weight attributes. Two types of evaluations were performed., and they were related to the set of similar cases (retrieve step) and to the suggested solution (reuse step).

Regardless of the application that is envisaged, most works reported in the literature evaluated their CBR for a given *k* using the suggested solution at the end of the reuse step. In our approach, the choice of the number of *k* similar cases is left to the user. Experimental tests showed that the results obtained with *H_WHSM* were weakly impacted by the *k* value. For instance, the retrieval of four similar cases rather than one has little influence on the suggested solution. However, it gives more information to the user about the coherence of the possible solutions.

The selection of relevant attributes and the weighting scheme was believed to have a significant influence on the similarity measure. To examine further the importance of using the CDT for the selection of attributes, we presented the results that were obtained with the similarity measure that do not propose the selection of relevant attributes. In most of the tests, *HEOM* [42] is the worst measure with respect to the true positive rate and the percentage of correct suggested solutions for all of the decisions. In addition, our results highlighted the importance of choosing a pertinent weighting scheme approach. Indeed, when comparing *GWHSM_GA* with *H_WHSM*, we observed that the weight had an impact on the determination of similar cases. *GWHSM_GA*, which used a learning-based approach with no clinical knowledge, has a lower sensitivity and specificity than *H_WHSM*. It is noted that the evaluation conditions were to the advantage of the GA weighting approach for the leave-one-out cross validation. Contrary to the deductive approach, it required a learning case-base, which was identical to the test case-base used to perform the leave-one-out cross validation. For the prosthesis choice, when the real case-base is the training set, *GWHSM_GA* retrieves 65% of cases as the correct solution when *k = 1* as opposed to 98% for the hierarchical metric. Even if more similar cases are retrieved (*k = 4*), the correct decision appears at least once in 95% of cases for *GWHSM_GA*, and reaches 100% for *H_WHSM*.

Using the CDT in the similarity measure enables us to select gradually relevant past cases, which is the key point of the hierarchical metric. This case selection allows the most relevant attributes to be indirectly weighted. The sensitivity and specificity of *H_WHSM* were better than those obtained for the two state-of-the-art similarity measures. We noted that for a few specific vascular accesses (trans-subclavian, trans-apical and trans-aortic), *HEOM* and *GWHSM_GA* had a low sensitivity value under 0.50. With these two similarity measures, the CBR mostly suggested the wrong solution for each case having these particular vascular accesses. With the hierarchical measure *H_WHSM*, the correct decision was suggested in more cases, even though only few cases with these vascular accesses were available in the case-base.

Although the proposed CBR, which was implemented using the *H_WHSM* based retrieve process, can be implemented with a small dataset, the information that is available in the case-base has an impact on the result. The issue related to the pre-processing (case maintenance) of the case-base has not been addressed in this work. In future work, the enrichment of the case-base will be considered.

The different results have shown that the selection of relevant attributes influenced the set of similar cases, and consequently, the suggested solution. This impact was also observed through the specificity and sensitivity of *H_WHSM* (Figs 7 and 8). Although the right and left trans-femoral accesses represented the majority of cases, their specificity was lower than that of the other accesses. The sensitivity reached 0.75 and 0.72 for the right and left trans-femoral accesses, respectively. With the attributes being clinically available, it was difficult for the different similarity measures to distinguish the right trans-femoral access from the left trans-femoral access. Only the clinical attributes related to the diameter of the femoral arteries were used in the case description to discern these two trans-femoral accesses. These attributes are ordinal data, and are known to be operator dependent. To better characterise cases, further quantitative attributes related to the tortuosity and the calcification of each femoral artery could be extracted from CT images for inclusion in the case-base. More generally, the CBR performance could still be improved by completing the case-base with additional relevant attributes, consistently with the CDT (e.g., patient's clinical history).

Even though the standard data available in the clinical routine are used in the proposed approach, the issue of missing information may be an issue. Thus, an incomplete description of cases could distort the results. There are different approaches to managing missing values. Although CBR already integrates a neutral approach in the similarity measure, the behaviour of the similarity measure when values are missing remains to be investigated. However, the amount of missing data can be easily quantified and integrated into the GUI to indicate to the user the data reliability of the retrieved cases.

## Conclusion

This study addressed the issue of case-based reasoning for the planning of TAVI procedures, focusing especially on decisions pertaining to the vascular access route and the valve prosthesis type. Special emphasis was placed on the retrieve and reuse steps. A new hierarchical similarity measure, that is based on clinical decision trees, was formulated to select and weight relevant attributes. Results show that the CBR performance is improved by considering a problem-specific similarity measure that integrates expert knowledge and reasoning.

The similarity measure could still be enhanced. Commonly available clinical attributes were used in the studied similarity measures. The evaluation of some relevant clinical attributes, such as tortuosity and calcification, may be operator-dependent, imprecise, or even missing. Pre-operative images or statistical shape models could be exploited to automatically extract additional high-level quantitative attributes, making them more sensitive and further improving the similarity measure.

## Supporting information

S1 Appendix State-of-the-art similarity measures HEOM and GWHSM. (DOCX)

S2 Appendix Clinical attributes of the case-base. (DOCX)

S1 File The global case-base. (CSV)
