# Supplementary material for: Similarity measures and attribute selection for case-based reasoning in transcatheter aortic valve implantation
Source: PLoS One. 2020 Sep 3;15(9):e0238463. doi: 10.1371/journal.pone.0238463 (PMC7470320; doi:10.1371/journal.pone.0238463)
Supplement: S1 Appendix — (DOCX) [file pone.0238463.s001.docx]

In this study, the hierarchical similarity measure is compared with two state-of-the-art similarity measures: *HEOM* and *GWHSM*, which is weighting with a genetic algorithm.

The *HEOM* is defined as follows:

| $HEOM\left( C_{c},C_{i} \right)=1-diss\left( C_{c},C_{i} \right)$ | (A1) |
| --- | --- |
| $diss\left( C_{c},C_{i} \right)= \sqrt{\sum_{a=a_{1}}^{a_{n}} d\left( C_{c,a},C_{i,a} \right)^{2}}$ |  |

where $d\left( C_{c,a},C_{i,a} \right)$ defines the heterogeneous function between the attribute $a$ of the two cases $C_{c}$ and $C_{i}$:

| $d\left( C_{c,a},C_{i,a} \right)= \left\{ \begin{matrix} overlap\left( C_{c,a},C_{i,a} \right), \\ diff\left( C_{c,a},C_{i,a} \right), \\ 1, \end{matrix} \right.\begin{matrix} if C_{c,a} and C_{i,a} are qualitative \\ if C_{c,a} and C_{i,a} are quantitative \\ if C_{c,a} or C_{i,a} is unknown \end{matrix}$ | | (A2) | |
| --- | --- | --- | --- |
| where | $overlap\left( C_{c,a},C_{i,a} \right)= \left\{ \begin{matrix} 0, \\ 1, \end{matrix}\begin{matrix} ifC_{c,a}=C_{i,a} \\ otherwise \end{matrix} \right.$ |  | |
| and | $diff\left( C_{c,a},C_{i,a} \right)= \frac{\left\vert C_{c,a}-C_{i,a} \right\vert}{{range}_{a}}$ |  |  |

Normalised attributes are used in the Euclidean distance function where the value ${range}_{a}={max}_{a}- {min}_{a}$. ${max}_{a}$ and ${min}_{a}$ represent, respectively, the possible maximum and minimum values of the attributes. If these values are unknown, they are fixed according to the training set.

The second similarity measure *GWHSM* uses different metrics for categorical attributes and numerical attributes as explained in the following equation.

| $GWHSM\left( C_{c},C_{i} \right)=1-diss\left( C_{c},C_{i} \right)$ | (A3) |
| --- | --- |
| $diss\left( C_{c},C_{i} \right)= \sqrt{\frac{\sum_{a=a_{1}}^{a_{n}} w_{a} {d\left( C_{c,a},C_{i,a} \right)}^{2}}{\sum_{a=a_{1}}^{a_{n}} w_{a}}}$ |  |
| With $d\left( C_{c,a},C_{i,a} \right)=\left\{ \begin{matrix} discard attribute \\ d_{C}\left( C_{c,a},C_{i,a} \right) \\ \left\vert C_{c,a}-C_{i,a} \right\vert\end{matrix} \begin{matrix} if C_{c,a} or C_{i,a} are unknown \\ if C_{c,a}and C_{i,a} are categorical \\ if C_{c,a} and C_{i,a} are numerical \end{matrix} \right.$ |  |
| Where $d_{C}\left( C_{c,a},C_{i,a} \right)=\left\{ \begin{matrix} 1 if C_{c,a}\neq C_{i,a} \\ 0 if C_{c,a}=C_{i,a} \end{matrix} \right.$ |  |
