# Supplementary material for: Similarity measures and attribute selection for case-based reasoning in transcatheter aortic valve implantation
Source: PLoS One. 2020 Sep 3;15(9):e0238463. doi: 10.1371/journal.pone.0238463 (PMC7470320; doi:10.1371/journal.pone.0238463)
Supplement: S2 Appendix — (DOCX) [file pone.0238463.s002.docx]

The following attributes are used in the case-based reasoning. They were extracted from the data base of the University Hospital of Rennes. Some of them are used in the similarity measure. The others, which provide additional information to the physicians about the procedure outcomes, are displayed in the graphical user interface.

| Attribute Label | Code/Unit/Comment |
| --- | --- |
| 1. Demographics |  |
| - Gender | {Male, Female} |
| - Age | [Years] |
| - Height | [cm] |
| - Weight | [kg] |
| - BSA | [m²] |
| - BMI | [kg/m²] |
| 1. Echocardiographic Measurements |  |
| - LVEF | [%] |
| - Aortic valve dPmean | [mmHg] |
| - Aortic valve dPmax | [mmHg] |
| - Aortic valve regurgitation | {0,1,2,3,4} |
| - Aortic valve area | [cm²] |
| - Aortic Annulus Diameter | [mm] |
| 1. CT Measurements |  |
| - Diameter Ascending Aorta | [mm] |
| - Minimal Diameter Aortic Annulus | [mm] |
| - Maximal Diameter Aortic Annulus | [mm] |
| - Calcification of the Aorta | {No, Mild, Moderate, Heavy, Massive} |
| - Calcification of the Valve | {No, Mild, Moderate, Heavy, Massive} |
| - Extension of the calcifications in the LVOT | True, false |
| - End diastolic interventricular septum thickness | [mm] |
| - Minimal diameter of the right iliac artery | [mm] |
| - Minimal diameter of the left iliac artery | [mm] |
| - Calcification of the femoral arteries | {No, Mild, Moderate, Heavy, Massive} |
| - Tortuosity of the femoral arteries | {No, Mild, Moderate, Severe} |
| - Minimal diameter of the left subclavian artery | [mm] |
| - Calcification of the left subclavian artery | {No, Mild, Moderate, Heavy, Massive} |
| - Tortuosity of the left subclavian artery | {No, Mild, Moderate, Severe} |
| - Mid-sinus diameter | [mm] |
| - Sinotubular junction diameter | [mm] |
| 1. Operative Data |  |
| - Surgery | {TAVI} |
| - Access | {trans-apical, left trans-femoral, right trans-femoral, trans-aortic, left trans-subclavian} |
| - Aortic valve size | [mm] |
| - Model of aortic valve | Free text (CoreValve, Edwards, etc.) |
| - Procedural Success | {true, false} |
| - TAVI coronary occlusion | {true, false} |
| - Cause of failure | Free text |
| - Procedural death | {true, false} |
| - Post Procedural Bleeding | {true, false} |
| - TAVI Annulus Rupture | {true, false} |
| - Revision Surgery | Free text |
| - Stroke Within 30days | {true, false} |
| - Prolonged Ventilation | {true, false} |
| - Prolonged Delirium | {true, false} |
